# Construction and Validation of an Immune Infiltration-Related Gene Signature for the Prediction of Prognosis and Therapeutic Response in Breast Cancer

**DOI:** 10.3389/fimmu.2021.666137

**Published:** 2021-04-27

**Authors:** Yang Peng, Haochen Yu, Yudi Jin, Fanli Qu, Haoyu Ren, Zhenrong Tang, Yingzi Zhang, Chi Qu, Beige Zong, Shengchun Liu

**Affiliations:** ^1^ Department of Endocrine and Breast Surgery, The First Affiliated Hospital of Chongqing Medical University, Chongqing, China; ^2^ Department of Breast Surgery, The Sixth Affiliated Hospital, Sun Yat-sen University, Guangzhou, China

**Keywords:** gene signature (GS), breast cancer, immune infiltration, immunotherapy, prognosis, chemotherapy

## Abstract

Breast cancer patients show significant heterogeneity in overall survival. Current assessment models are insufficient to accurately predict patient prognosis, and models for predicting treatment response are lacking. We evaluated the relationship between various immune cells and breast cancer and confirmed the association between immune infiltration and breast cancer progression. Different bioinformatics and statistical approaches were combined to construct a robust immune infiltration-related gene signature for predicting patient prognosis and responses to immunotherapy and chemotherapy. Our research found that a higher immune infiltration-related risk score (IRS) indicates that the patient has a worse prognosis and is not very sensitive to immunotherapy. In addition, a new nomogram was constructed based on the gene signature and clinicopathological features to improve the risk stratification and quantify the risk assessment of individual patients. Our study might contribute to the optimization of the risk stratification for survival and the personalized management of breast cancer.

## Introduction

Breast cancer remains a major threat to women’s health and wellness. It is the leading cause of cancer-related deaths among females worldwide (1). Advances in early detection and treatment have greatly reduced breast cancer mortality, but patients still succumb to the development of metastasis from breast cancer. These sobering data indicate an urgent need for innovative approaches to breast cancer treatment based on chemotherapy and hormonal therapy to reduce disease recurrence and death from this disease. In recent years, increasing research has supported the important role of the immune system in tumorigenesis and cancer development. Likewise, the immune system plays a key role in determining the occurrence of breast cancer and death, the response to standard therapy and the long-term survival of breast cancer patients (2). The current success of antagonists targeting immune checkpoints in a variety of solid tumors (3, 4) has rekindled interest in immunotherapy-based therapies for the treatment and prevention of breast cancer (4, 5).

The immune system consists of various proteins, immune cells and tissues with a complex structure and is an important means of host defense (6). Tumor-infiltrating immune cells are the main immune signature and are strongly associated with the clinical outcome of immunotherapy (7). T cells play a pivotal role in the initiation, progression and treatment of cancer (especially immunotherapy) (8). There are two major classes of T cells, namely, CD4+ T cells and CD8+ T cells, each of which includes many functional subpopulations. Importantly, the abundance of T cell subsets, especially tumor-infiltrating T cells, can influence clinical outcomes and prognosis (9). Therefore, the investigation of immune cell characteristics can lead to a better understanding of the interactions between the immune system and tumors and provide important clues to improve the development of immunotherapy in precision medicine (10).

Two-thirds of the genome is active in one or more immune cell types, and less than 1% of genes are uniquely expressed in a given cell type. Therefore, it is crucial that the expression patterns of these immune cell types are decoded in the context of a network rather than as individuals for their roles to be properly characterized and interrelated (11). Gene expression analysis is useful for analyzing defects in the immune system, such as autoimmune diseases, immunodeficiency viruses and malignancies. For example, an analysis of the systematic variation in gene expression can correlate expression patterns with specific diseases and identify gene networks important for targeting immune function (12). Therefore, establishing the relationship between gene expression networks and the immune system may provide new clues for the immunotherapy and prognosis of malignant tumors.

Since the introduction of first-generation multigene assays, several prognostic assays for breast cancer, such as Prosigna (13) and EndoPredict (14), have subsequently been developed. The most commonly used gene signature in clinical work is a 21-gene (16 cancer-related genes and 5 reference genes) signature assay based on quantitative real-time reverse transcription PCR (qRT-PCR) (15). Finak *et al.* found a new 26-gene stroma-derived prognostic predictor (SDPP) associated with the clinical outcome of breast cancer patients. These 26 genes comprise several genes that are closely related to the immune response (16). Thus, exploring the immune gene network associated with breast cancer is of great importance for the precise treatment of breast cancer. However, such studies have not been reported very often.

In our study, we first identified the immune cells associated with breast cancer. Then, weighted gene coexpression network analysis (WGCNA) was applied to identify the genes that are highly associated with these immune cells. After building a prediction model with these genes, we found that the model has great predictive power and can be validated in a variety of other datasets. It is also predictive of responses to chemotherapy and immunotherapy. This model provides clinicians with a novel and powerful reference for diagnosis and treatment.

## Material and Methods

### Dataset Preparation And Data Processing

The infiltration scores of 24 immune cells were obtained from the Immune Cell Abundance Identifier (ImmuCellAI) (http://bioinfo.life.hust.edu.cn/web/ImmuCellAI/) (17) and normalized using the scale method in R software (version 4.0.3; http://www.r-project.org). The RNA sequencing (RNA-seq) data generated from the Illumina HiSeq RNA-Seq platform and corresponding clinical information of 1007 breast cancer patients were downloaded from The Cancer Genome Atlas (TCGA) (https://gdc-portal.nci.nih.gov/) and used as the training set. The gene expression information of 1761 patients was downloaded from the Molecular Taxonomy of Breast Cancer International Consortium (METABRIC) database (https://www.cbioportal.org/study?id=breastcancer_metabric) and used as the first validation set. The clinical information distribution of the TCGA and METABRIC cohorts is shown in **Supplemental Table S1**. In addition, the GSE20685 (n = 327) and GSE21653 (n = 266) datasets, which contain gene expression matrix files from the Gene Expression Omnibus (GEO) database (https://www.ncbi.nlm.nih.gov/geo/) and are both based on the GPL570 platform, were used as another two external validation sets. Only patients with overall survival (OS) times of more than 30 days were included in all datasets. In addition, gene-level proteomics data were downloaded from the Clinical Proteomic Tumor Analysis Consortium (CPTAC) website (https://proteomics.cancer.gov/programs/cptac).

### Study Design

A univariate Cox proportional hazards (Cox-PH) regression model was performed to identify significant prognosis-related infiltrating immune cells in breast cancer using the “survival” R package (version: 3.2-7; https://cran.r-project.org/web/packages/survival/index.html). Then, 11 prognosis-related infiltrating immune cells were found.

WGCNA is a systematic analysis tool that describes patterns of correlation between genes in different microarray samples and clusters genes into modules to investigate associations between genomes and clinical traits. The first step was to calculate the correlation coefficient (Pearson coefficient) between any two genes. To measure whether two genes have similar expression patterns, it is generally necessary to set a threshold value for screening, and those above the threshold value are considered similar. However, if the threshold is set to 0.8, it is difficult to show that 0.8 and 0.79 are significantly different from each other. Therefore, WGCNA uses weighted values of correlation coefficients, i.e., taking N powers of the gene correlation coefficients, so that the connections between genes in the network obey a scale-free network distribution (scale-free networks), and this algorithm is more biologically meaningful. The second step constructs a hierarchical clustering tree by correlation coefficients between genes, different branches of the clustering tree represent different gene modules, and different colors represent different modules. Based on the weighted correlation coefficients of genes, the genes are classified according to expression patterns, and genes with similar patterns are grouped into one module. In this way, tens of thousands of genes can be divided into dozens of modules by gene expression patterns, which is a process of extracting inductive information. In our research, with a larger R-squared and smaller mean connectivity to ensure a scale-free coexpression network, the soft threshold power was set to 12 (**Supplemental Figure S1**), and a total of 24 WGCNA nongray modules were identified. WGCNA was performed using the “WGCNA” R package (https://cran.r-project.org/web/packages/WGCNA/index.html) (18) to find the module that was most associated with these prognosis-related infiltrating immune cells. Module membership (MM) indicated the correlation between module eigengenes and gene expression profiles. Subsequently, we performed univariate analysis for each of the 802 genes in the red module. With a p value threshold of <0.05 in univariate Cox regression, 193 candidates that were significantly related to prognosis were identified.

Furthermore, least absolute shrinkage and selection operator (LASSO) Cox regression was used to further narrow down the range of candidate immune-related prognostic biomarkers using the “glmnet” R package (version: 4.0-2; https://cran.r-project.org/web/packages/glmnet/index.html). Fifteen immune-related genes were identified to have nonzero coefficients in the model, and the samples were separated into high- and low-risk groups based on the optimal cutoff value of -0.03, which was derived from the *surv_cutpoint* function of the “*survminer”* R package (Version: 0.4.3; https://cran.r-project.org/web/packages/survminer/index.html). The optimal cutoff value of -0.03 will also be applied to all datasets in our study. This includes the training set (TCGA) and the three validation sets (METABRIC, GSE21653 and GSE20685). The immune-related risk score (IRS) was established as follows:

IRS=sum of coefficients×normalized mRNA expression of immune‐related genes.

### Estimation of Immunotherapy and Chemotherapy Responses

Six immune infiltration cell scores and 28 immune infiltration cell scores were downloaded from the Tumor Immune Estimation Resource (TIMER) database (available at http://cistrome.org/TIMER) (19) and The Cancer Immunome Atlas (TCIA) database (https://tcia.at/), respectively (20). The estimated scores, immune scores and stromal scores of breast cancer were analyzed by the “estimate” R package, which provides researchers with scores for tumor purity, the level of stromal cells present, and the infiltration level of immune cells in tumor tissues based on expression data (21). Immunotherapy response (anti-PD1 or anti-CTLA4 therapy) data from TCGA were downloaded from ImmuCellAI, a gene set signature-based method that was built for predicting the immunotherapy response with high accuracy (area under curve 0.80–0.91) by analyzing gene expression data (17). Therefore, the methods provided by ImmuCellAI were used to predict the sensitivity of immunotherapy for each breast cancer patient from the TCGA database. Patients were divided into a response group and a nonresponse group. Then, we applied chi-square test analysis between the immunotherapy response group and IRS group and found that the percentage of the immunotherapy response group in the low-IRS group was much higher than that in the high-IRS group (P <0.001).

The drug sensitivity data of cancer cell lines (CCLs) were obtained from the Cancer Therapeutics Response Portal (https://portals.broadinstitute.org/ctrp), which contains sensitivity data for 481 compounds in 835 CCLs, and the PRISM Repurposing dataset (19Q4, released December 2019, https://depmap.org/portal/prism/), which contains sensitivity data for 1448 compounds in 482 CCLs. Both datasets present the area under the dose–response curve (area under the curve—AUC) value as a measure of drug sensitivity, with lower AUC values indicating higher sensitivity to treatment. The drug sensitivity of each sample from TCGA was assessed by the R package *pRRophetic* (Version: 4.15-1; https://github.com/paulgeeleher/pRRophetic), which has a built-in ridge regression model that was used to predict the chemotherapy response of clinical samples based on their expression profiles, resulting in an AUC estimate for each compound in each clinical sample from TCGA (22) (23). Then, the AUC values and IRS were analyzed by Spearman correlation analysis to select compounds with negative correlation coefficients (Spearman r < −0.25 for CTRP or −0.30 for PRISM). Next, an analysis of differences in drug response between the high IRS score (highest decile) and low IRS score (lowest decile) groups was performed to identify compounds with lower estimated AUC values in the high-IRS group (log2FC>0.05).

### Bioinformatic and Statistical Analyses

All statistical analyses were performed using R version 4.0.3 (2020-10-10). Gene set enrichment analysis (GSEA) was performed to validate the immune status of the high-IRS group with the “c5.go.v7.2.entrez.gmt” gene set using the “clusterProfiler” R package (Version: 3.18.0; https://bioconductor.org/packages/clusterProfiler/) (24). The Kaplan–Meier method was used to draw survival curves, and the log-rank test was used to evaluate significant differences (P < 0.05). Time-dependent receiver operating characteristic (tROC) analysis was used to measure the predictive power of the model with the “timeROC” R package (Version: 0.4; https://cran.r-project.org/web/packages/timeROC/index.html). A meta-analysis was performed to evaluate the prognostic value of the gene signature in all datasets using the “meta” R package (Version: 4.15-1; https://cran.r-project.org/web/packages/meta/index.html). The “rms” R package was used to plot the nomogram and the calibration curve. The “ggplot2” R package (Version: 3.3.2; https://cran.r-project.org/web/packages/ggplot2/index.html) was used to plot the correlation bubble diagram. P < 0.05 (two-sided) was considered statistically significant.

## Results

### Schematic Diagram of the Study Design

**Figure 1:**
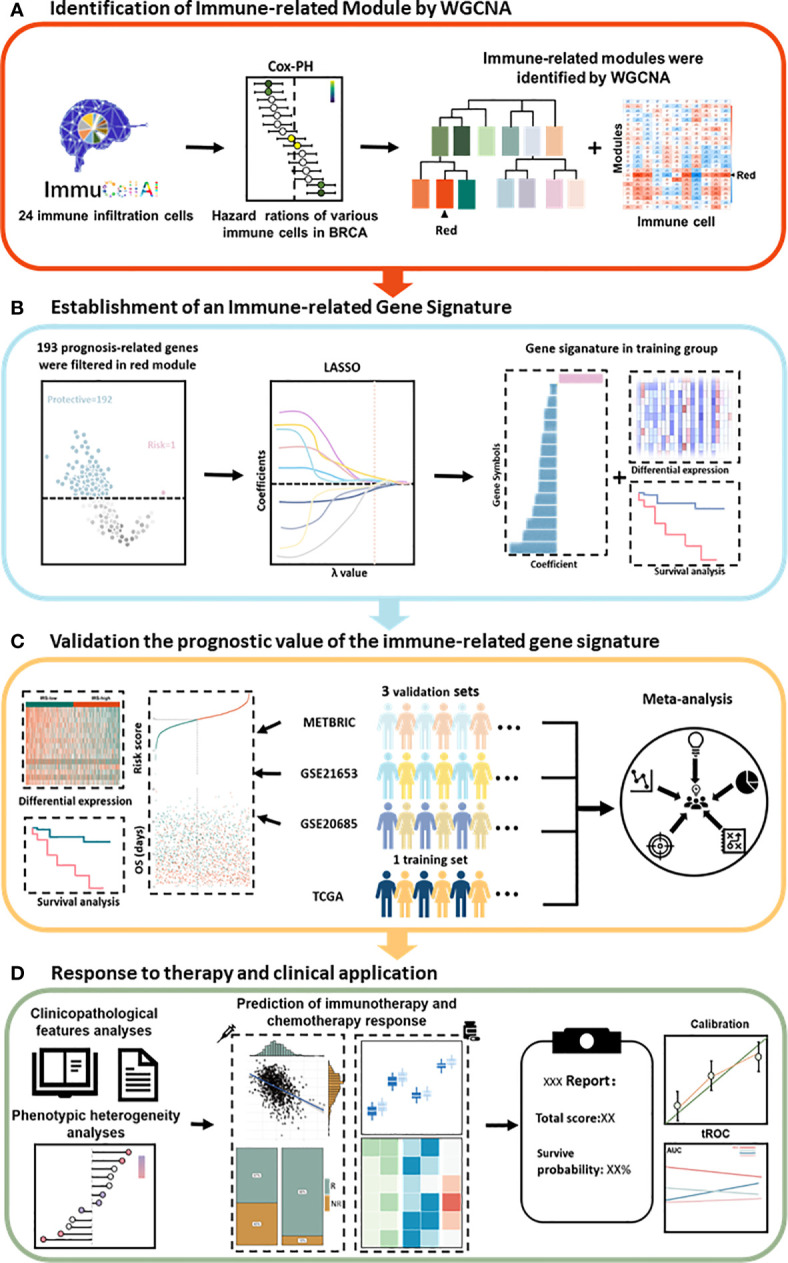
Schematic diagram of the study design. **(A)** The red module was identified as the immune-related module in breast cancer by WGCNA. **(B)** Combined methods were used to establish a robust immune-related gene signature for prognosis. **(C)** The prognostic value of the gene signature was validated in different cohorts. **(D)** Clinical features and application of the IRS. Cox-PH, Cox proportional hazards; LASSO, least absolute shrinkage and selection operator; tROC, time-dependent receiver operating characteristic; WGCNA, weighted gene coexpression network analysis.

First, the infiltration scores of 24 immune cells were obtained from ImmuCellAI and normalized using the scale method in R language software. Univariate Cox regression analysis identified immune cells that play a protective role in breast cancer patients. WGCNA identified gene modules associated with protective immune cells (**Figure 1A**). Then, a total of 193 candidate genes, which were most associated with prognosis, were identified from the most immune-related WGCNA module (red module). The LASSO algorithm was used to identify promising candidates and establish a robust signature containing 15 immune-related genes to predict survival (**Figure 1B**). Subsequently, the prognostic value of this gene signature was evaluated in the TCGA training set and in the three independent external validation sets. In addition, a meta-analysis was performed to further validate its predictive power (**Figure 1C**). A good signature should be able to be applied clinically. Therefore, we analyzed the clinicopathological features and predicted the response to immunotherapy of patients in different risk groups. We also predicted the choice of chemotherapeutic drugs considering the tumor heterogeneity of patients in different risk groups. Finally, a nomogram was built based on the IRS and other clinicopathological variables to quantify the risk assessment and survival probability of breast cancer patients (**Figure 1D**).

### Establishment of an Immune-Related Gene Signature for Prognosis

**Figure 2:**
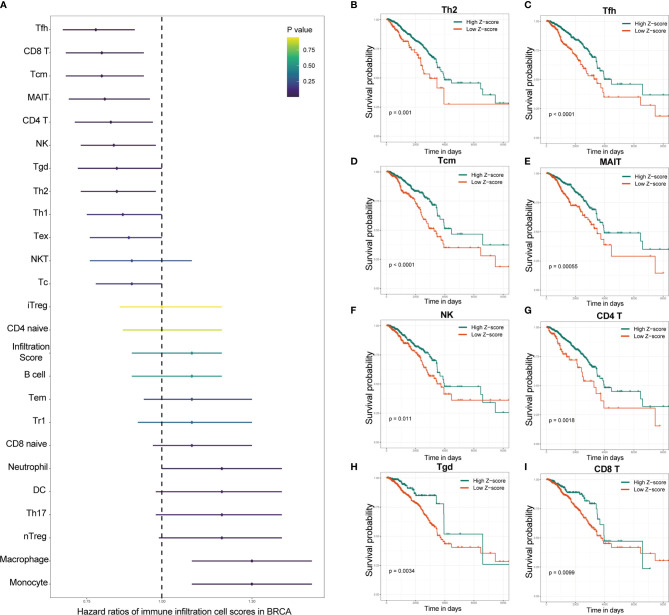
Specific immune cells were identified as protective factors for survival by the immune infiltration score. **(A)** Univariate Cox regression analysis showed that several of the various immune cells in breast cancer were major protective factors. Kaplan–Meier analysis showed that patients with higher immune infiltration scores of Th2 **(B)**, Tfh **(C)**, Tcm **(D)**, MAIT **(E)**, NK **(F)**, CD4 T **(G)**, Tgd **(H)** and CD8 T **(I)** cells exhibited better OS. OS, overall survival; IRS, immune infiltration-related score. NK cells, natural killer cells; Th2 cells, T helper 2 cells; Tfh cells, T helper cells; MAIT cells, mucosal-associated invariant T cells.

**Figure 3:**
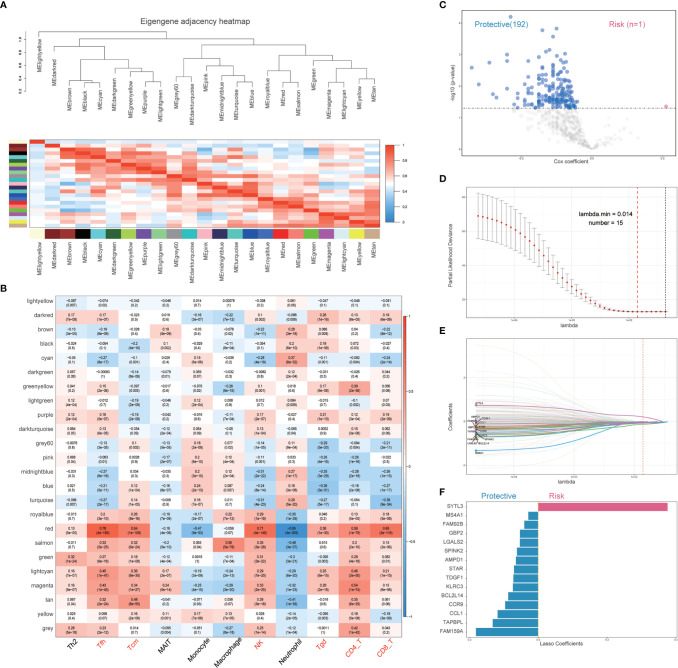
Establishment of an immune-related gene signature with WGCNA. **(A)** Clustering diagram of the correlation among the 24 WGCNA modules. Correlation value is color coded. Red indicates a positive correlation, blue indicates a negative correlation, and the degree of color is proportional to the correlation. **(B)** Correlation heatmap between WGCNA modules and immune cell infiltration scores. (r values and p-values are shown; correlation values are color-coded). **(C)** Association between red-module genes and survival. The Cox coefficient and p-value of each gene are shown. Only genes with p<0.05 were considered for further analysis. A total of 193 promising candidates were identified among the hub genes extracted from the red module. Blue markers indicate prognostic protective genes, while red markers indicate the opposite. **(D)** The adjustment parameter (lambda) in the LASSO model was selected for 10-fold cross-validation by the minimum criterion. Partial likelihood deviation curves were plotted against lambda. Dotted vertical lines were drawn at the optimal values by using the minimum criterion and 1 standard error of the minimum criterion (1-SE criterion). **(E)** LASSO coefficient profiles of the 193 immune-related genes. A coefficient profile plot was produced against the log (lambda) sequence. A vertical line was drawn at the value selected using 10-fold cross-validation, where the optimal lambda resulted in 15 nonzero coefficients. **(F)** Distribution of the LASSO coefficients of the 15 immune-related gene signatures. The horizontal coordinate indicates LASSO coefficients, genes with negative coefficients in this regression indicate prognostic protective genes (blue marker), and positive numbers indicate poor prognostic genes (red marker). LASSO, least absolute shrinkage and selection operator; WGCNA, weighted gene coexpression network analysis.

Univariate Cox regression analysis based on immune infiltration scores was performed, and the results were ranked to identify several immune cells in breast cancer that are the main protective factors, including Tfh, CD8 T, Tcm, MAIT, CD4 T, NK, Tgd and Th2 cells (**Figure 2A**). Kaplan–Meier analysis showed that patients with higher immune infiltration scores exhibited better OS (**Figures 2B–I**). WGCNA is a systematic biological method used to describe gene association modules between different samples and can be used to identify highly synergistic sets of genes. WGCNA was used to analyze whole-transcriptome profiling data and immune infiltration scores (**Figure 3A**). With a larger R-squared value and a smaller mean connectivity value to ensure a scale-free coexpression network, the soft threshold power was set to 12 (**Supplemental Figure S1**), and a total of 24 nongray WGCNA modules were identified. Among these modules, the red module correlated with the infiltration scores of six immune cells that had been identified as protective factors for breast cancer (**Figure 3B**). Moreover, we performed GO analysis on 802 genes in the red module and found that gene enrichment in the red module was significantly enriched in the ‘T cell activation’, ‘regulation of T cell activation’, and ‘immune receptor activity’ terms based on GO analysis (**Supplemental Figure S2**). Therefore, the red module was identified as the immune-related module. Subsequently, we performed univariate analysis for each of the 802 genes in the red module. With a p value threshold of <0.05 in univariate Cox regression, 193 candidates (192 protective markers and 1 risk marker) that were significantly related to prognosis were identified (**Figure 3C**). The LASSO Cox regression model was then used to identify the genes with the most robust prognostic value. Tenfold cross-validation was applied to overcome overfitting, with an optimal λ value of 0.014 selected (**Figure 3D**). Finally, 15 candidate genes (SSYTL3, MS4A1, FAM92B, GBP2, LGALS2, SPINK2, AMPD1, STAR, TDGF1, KLRC3, BCL2L14, CCR9,CCL1, TAPBPL and FAM159A) were identified to have nonzero LASSO coefficients and were included in the gene signature model (**Figure 3E** and **Supplemental Table S2**). The distribution of the LASSO coefficients of the genes in the signature is shown in **Figure 3F**.

### Prognostic Analysis of the Immune-Related Gene Signature in the Training Cohort

**Figure 4:**
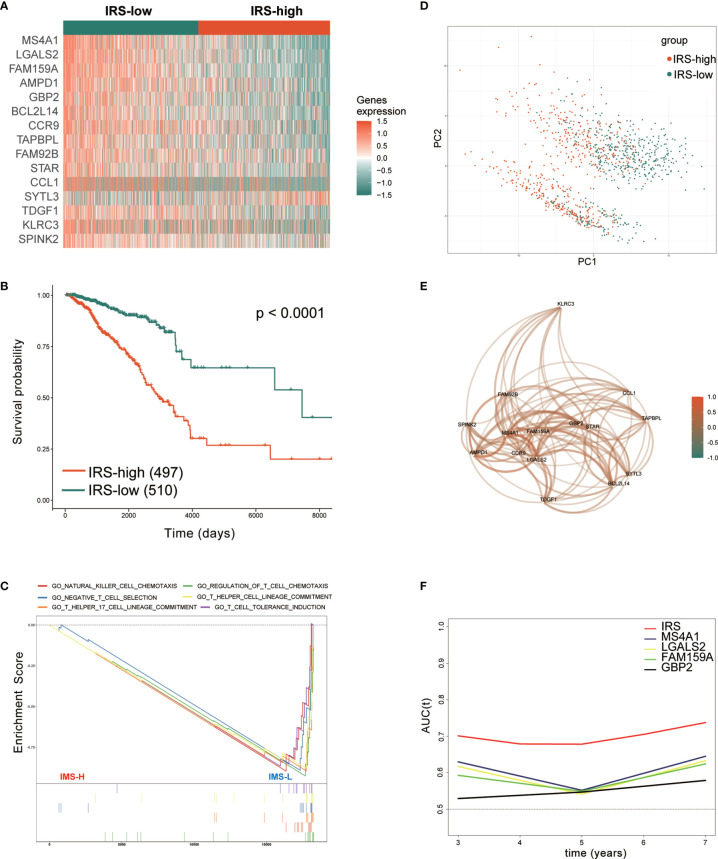
Prognostic analysis of the immune-related gene signature in the training set. **(A)** Heatmap of the 15 immune-related prognostic genes between the high- and low-IRS groups in the training set. **(B)** Kaplan–Meier curves identified that patients in the high-IRS group exhibited better OS in the training set. **(C)** GSEA showed that several immune-related signaling pathways were inhibited in the high-IRS group. **(D)** PCA plot between the high- and low-IRS groups in the training set. **(E)** The correlation network of candidate genes. Correlation coefficients are shown in different colors. **(F)** tROC analysis showed that the IRS was an accurate variable for survival prediction. The four genes shown in the figure have the four highest AUCs among the 15 signature genes. TCGA, The Cancer Genome Atlas; GSEA, gene set enrichment analysis; HR, hazard ratio; IRS, immune-related risk score; High-IRS, high immune-related risk score; Low-IRS, low immune-related risk score; OS, overall survival; tROC, time-dependent receiver operating characteristic.

First, the IRS was established as described in the methods section. Then, we performed prognostic analysis of the IRS genes in the TCGA training set. The 1007 TCGA samples were separated into high- and low-risk groups based on the optimal cutoff value of -0.03. The expression of 15 genes in the gene signature was further analyzed. Fourteen genes in the low-IRS group showed higher expression than those in the high-IRS group, except for SYTL3, which is thought to be a risk gene (**Figure 4A**). This finding is consistent with the previous results. Kaplan-Meier curves showed that patients in the high-IRS group exhibited worse OS in the training group (P < 0.001, **Figure 4B**). Looking further into the cause, with the “c5.go.v7.2.entrez.gmt” gene set from the Molecular Signatures Database (MSigDB), **Figure 4C** demonstrates that IRS groups have different immune populations. Enrichment of NK and T cell chemotaxis and commitment genes in IRS-low tumors. (**Figure 4C**). As shown in **Figure 4D**, principal component analysis (PCA) showed that patients in different risk groups were mainly distributed in two directions. Correlation analysis revealed that these 15 genes were strongly correlated with each other (**Figure 4E**). Furthermore, tROC analysis showed the AUCs of the IRS and four genes. These four genes had the highest AUCs among the 15 signature genes. (**Figure 4F**). This also indicated that the IRS was the most accurate predictor of OS compared with a single gene. To prove the applicability of IRS in clinical work. We analyzed the association of IRS with clinicopathological features and clinical information. It is also evident in **Table 1** that the different IRSs are inextricably linked to the clinicopathological features and basic clinical information of the patients.

**Table 1:** The association between IRS and pathological features.

	IRS-high (N=478)	IRS-low (N=497)	P-value
Age^****^			
Mean (SD)	60.9 (13.2)	56.1 (13.0)	<0.001
Median [Min, Max]	61.0 [29.0, 90.0]	55.0 [26.0, 90.0]	
Menopausal State^****^			
Post	335 (70.1%)	295 (59.4%)	<0.001
Pre	143 (29.9%)	202 (40.6%)	
Histology Subtype^****^			
Ductal/NST	369 (77.2%)	329 (66.2%)	<0.001
Other	109 (22.8%)	168 (33.8%)	
**Tumor Stage**			
I	72 (15.1%)	99 (19.9%)	0.053
II	276 (57.7%)	281 (56.5%)	
III	109 (22.8%)	110 (22.1%)	
IV	12 (2.5%)	4 (0.8%)	
Missing	9 (1.9%)	3 (0.6%)	
ER Status^**^			
Negative	83 (17.4%)	119 (23.9%)	0.015
Positive	375 (78.5%)	360 (72.4%)	
Missing	20 (4.2%)	18 (3.6%)	
**PR Status**			
Negative	150 (31.4%)	147 (29.6%)	0.558
Positive	308 (64.4%)	331 (66.6%)	
Missing	20 (4.2%)	19 (3.8%)	
HER2 Status^***^			
Negative	312 (65.3%)	354 (71.2%)	0.002
Positive	92 (19.2%)	58 (11.7%)	
Missing	74 (15.5%)	85 (17.1%)	
Survival time^*^			
Mean (SD)	3.30 (3.17)	3.80 (3.25)	0.017
Median [Min, Max]	2.20 [0.0861, 23.3]	2.77 [0.0944, 23.9]	
Survival state^****^			
Alive	381 (79.7%)	464 (93.4%)	<0.001
Dead	97 (20.3%)	33 (6.6%)	

ER, estrogen receptor; PR, progesterone receptor.

*means p<0.05; **means p<0.01; ***means p<0.005; ****means p<0.001.

### Prognostic Validity of the Signature of 15 Immune-Related Genes in Breast Cancer

**Figure 5:**
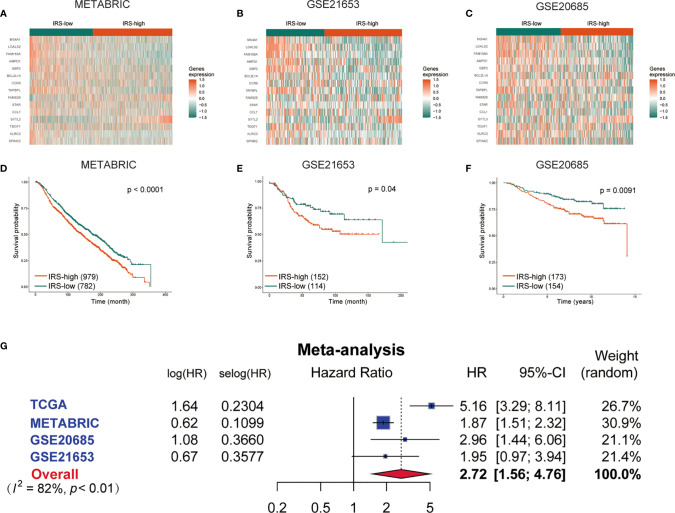
Validation of the gene signature in the external validation sets. The expression heatmap of the 15 immune-related prognostic genes between the high- and low-IRS groups in the METABRIC **(A)**, GSE21653 **(B)** and GSE20685 **(C)** cohorts. Kaplan-Meier curves for the OS of patients in the high-risk group and low-risk group in the METABRIC **(D)**, GSE21653 **(E)** and GSE20685 **(F)** cohorts. **(G)** Meta-analysis of the TCGA training set and 3 external validation sets. METABRIC, Molecular Taxonomy of Breast Cancer International Consortium; GSE, gene expression omnibus series; IRS, immune-related risk score; High-IRS, high immune-related risk score; Low-IRS, low immune-related risk score.

To confirm the stability and generalizability of the immune-related gene signature in different series, it was further confirmed in 3 independent external validation sets. The patients in the METABRIC, GSE21653 and GSE20685 cohorts were also assigned to either the high- or low-IRS group with the same calculation formula as that used for the TCGA cohort. The cutoff value in all three validation cohorts was -0.03, which was consistent with the training cohort. The gene expression heatmaps are displayed in **Figures 5A–C**. The analysis results of all three external validation sets were highly consistent with those of the training set. Kaplan–Meier analysis demonstrated that high IRS predicted worse OS than low IRS in the 3 validation cohorts (P < 0.0001, **Figure 5D**; P =0.04, **Figure 5E**; P =0.0091, **Figure 5F**). A meta-analysis was performed to analyze the gene signature in the pooled cohort integrating the training cohort and three validation cohorts, which were divided into two groups. As shown in **Figure 5G**, the meta-analysis demonstrated that all patients with a higher IRS exhibited a worse prognosis than those with a lower IRS (overall HR = 2.72, 95% CI 1.56–4.76). Finally, the correlation between the IRS signature and the 8 immune populations was verified again in two publicly available databases, TCGA and METABRIC. A negative correlation was found between IRS and most of the immune populations. (**Supplementary Figure S3**).

### Estimation of the Independent Prognostic Value of the Signature Containing 15 Immune-Related Genes

**Figure 6:**
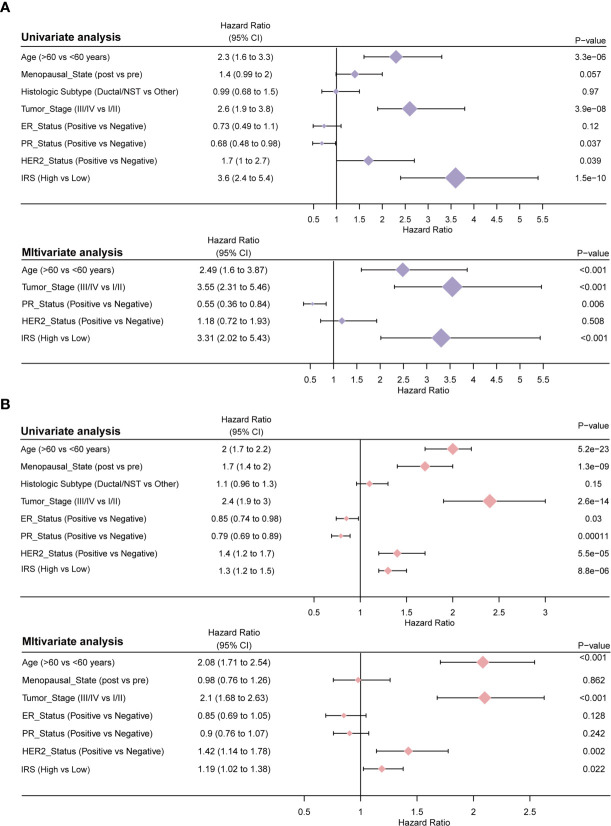
Results of the clinicopathological feature univariate and multivariate Cox regression analyses of OS in the TCGA training cohort **(A)** and the METABRIC validation cohort **(B)**.

Univariate Cox regression and multivariate Cox regression of the signature of the 15 IRS genes were performed in the TCGA dataset (P =1.5e-10, univariate Cox regression; P < 0.001, multivariate Cox regression, **Figure 6A**). The independence of the clinical prognostic significance of the signature in breast cancer was verified. The risk score showed significance in both univariate Cox regression and multivariate Cox regression. These consistent results were also validated in the METABRIC cohort (P =8.8e-06, univariate Cox regression; P =0.022, multivariate Cox regression, **Figure 6B**). The heterogeneity of breast cancer is very obvious. In particular, the expression of molecules between different subtypes is also different. Therefore, we reanalyzed the predictive power of IRS in different subtypes of breast cancer. **Supplementary Figure S4** (P =0.00026, univariate Cox regression; P =0.002, multivariate Cox regression, **Figure 6B**), S5 (P =0.016, univariate Cox regression; P =0.025, multivariate Cox regression) and S6 (P =0.017, univariate Cox regression; P =0.05, multivariate Cox regression) show univariate and multivariate analyses of the luminal, TNBC and HER2 subtypes, respectively. The results showed that in different subtypes, IRS was also statistically significant. The data in **Supplementary Figures S4–S6** are all from the TCGA database. It is worth noting that the 50-gene signature test (PAM50) was performed to identify breast cancer subtypes (13).

### Heterogeneity Between IRS-High and IRS-Low Patients

**Figure 7:**
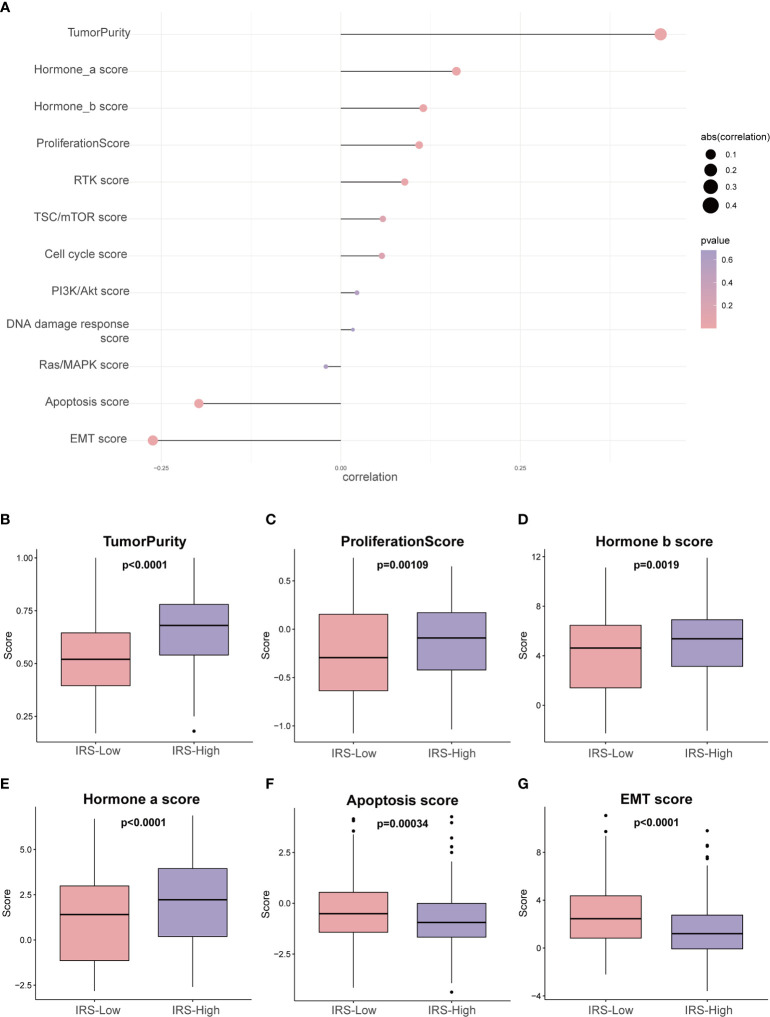
Phenotype heterogeneity between the high- and low-IRS groups in the training set. **(A)** The bubble map shows the correlation between the IRS and RPPA data-based scores. Boxplots show differences in **(B)** tumor purity, **(C)** proliferation, **(D)** hormone_b, **(E)** hormone_a, **(F)** apoptosis, and **(G)** EMT. The Kruskal–Wallis test was performed to calculate the P-value. IRS, immune-related risk score; RPPA, reversed-phase protein array; EMT, epithelial–mesenchymal transition.

To further investigate the tumor heterogeneity between the two groups of patients and to delve into the differences in tumorigenesis mechanisms between the two groups, reversed-phase protein arrays (RPPAs) were used to analyze the major pathways in the two groups. Through correlation analysis, we found that the IRS was significantly positively correlated with tumor purity (r = 0.45, P <0.01), the hormone a score (r = 0.16, P <0.01), the hormone b score (r = 0.12, P <0.01) and the proliferation score (r = 0.11, P <0.01) and significantly negatively associated with the EMT score (r = -0.26, P <0.01) and the apoptosis score (r = -0.20, P <0.01) (**Figure 7A** and **Supplemental Table S3**).

In addition, we sought to investigate whether the above pathway scores showed differences between the high- and low-IRS groups with breast cancer (**Figures 7B–G**). The pathway scores, which are protein expression signatures of pathway activity, associated with tumor lineage (**Figures 7B–G**) were from an RPPA as published by TCGA (25). Our analysis implies that the pathway scores for tumoricity, proliferation, hormone-b and hormone-a were significantly higher in the IRS-High group than in the IRS-Low group. On the other hand, the pathway scores for EMT and apoptosis were lower in the IRS-High group. These results suggest that the immune-related gene signature shows differences in most cancer-associated phenotypes.

The percentage of tumor cells in the tumor tissue is the tumor purity, so higher tumor purity predicts a worse prognosis, and IRS-High also predicts a worse prognosis, so they have a very significant correlation (**Figure 7A**). In addition, the expression levels of protective genes were lower in the IRS-high group, while the opposite was observed for risk genes, resulting in a poorer prognosis for high IRS. During tumor progression, cancer cells that are prone to proliferate and not readily undergo apoptosis usually result in a poorer prognosis. Such a speculation is consistent with the results we obtained (**Figures 7B, F**). The different dependence on hormones may be due to the heterogeneity of breast cancer itself. It is well known that high levels of estrogen have a stimulating effect on the progression of breast cancer (**Figures 7D, E**). Interestingly, the EMT pathway is usually associated with tumor invasion and metastasis, and tumors with active EMT are usually considered to have a relatively poor prognosis. This is contrary to our **Figure 7G** results and needs to be explored further.

### The Gene Signature Serves as a Valuable Marker for Immune Targets and Immunotherapy Response

**Figure 8:**
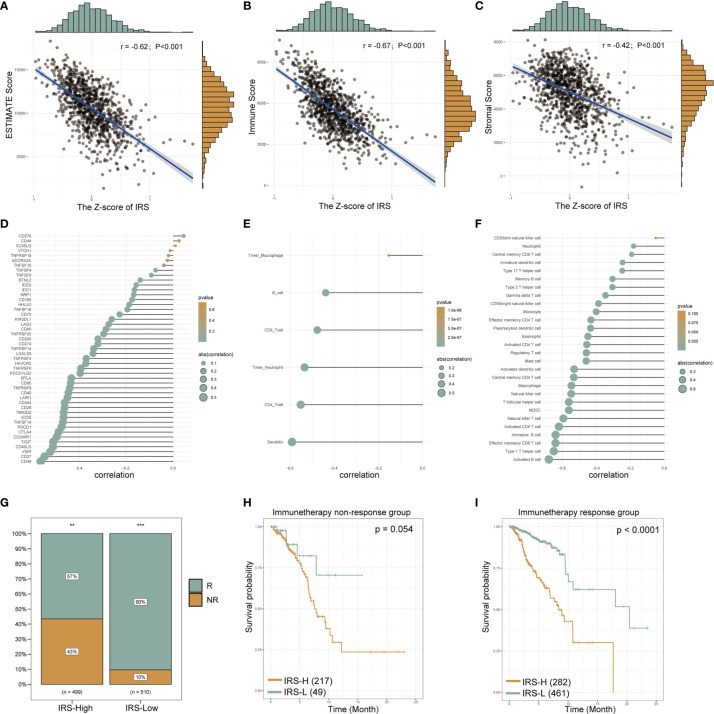
The gene signature serves as a valuable marker for immune targets and immunotherapy response. The estimated scores, immune scores and stromal scores of breast cancer from TCGA database were analyzed by the “estimate” R package. The correlation line chart shows the relationship between the Z-score of the IRS and the estimated score **(A)**, immune score **(B)** and stromal score **(C)** (r values and p-values are shown; a larger slope of the line in the graph means a higher correlation) (details see methods section). The correlation bubble chart shows the association between the Z-score of the IRS and immune checkpoint genes **(D)**, the 6 immune infiltration cell scores from TIMER **(E)** and 28 immune infiltration cell scores from TCIA **(F)** (see the *Methods* section for data acquisition). The size of the bubble represents the value of the correlation, and the shade of the color represents the value of the p-value. Proportion of immune-responsive and nonimmune-responsive populations in the IRS high- and low-expression groups **(G)**. Survival analysis graph showing differences in survival between IRS-high and IRS-low groups in nonimmune-responsive **(H)** and immune-responsive **(I)** populations. IRS, immune-related risk score; High-IRS, high immune-related risk score; Low-IRS, low immune-related risk score. **means p <0.01, ***means p<0.001.

Next, we sought to identify immunotherapy targets and assess the immunotherapy response in patients in the high- and low-IRS groups. The correlation analysis showed that the estimated score (r = -0.62; P < 0.001, **Figure 8A**), immune score (r = -0.67; P < 0.001, **Figure 8B**) and stromal score (r = -0.42; P < 0.001 **Figure 8C**) were negatively correlated with the Z-score of the IRS. Most of the common immune checkpoint genes were negatively correlated with the Z-score of the IRS (**Figure 8D** and **Supplemental Table S4**). The correlation analysis bubble diagram shows that the relationships among the 6 immune infiltration cell scores from TIMER (**Figure 8E** and **Supplemental Table S5**) and 28 immune infiltration cell scores from TCIA (**Figure 8F** and **Supplemental Table S6**) were negatively correlated with the Z-score of the IRS. This finding suggests that low-IRS patients may have more options for immune targets when selecting immunotherapy. **Figure 8G** shows the prediction of response to immunotherapy for patients in the IRS-High and IRS-Low groups. In the IRS-High group, 57% of the patients were predicted to respond to immunotherapy, but in the IRS-Low group, this percentage was 90%. Based on these data, we speculate that patients in the IRS-Low group may be more sensitive to immunotherapy. When survival analysis was performed in the immunotherapy responsive and nonresponsive groups separately, IRS levels were found to remain predictive of prognosis in both groups (**Figures 8H, I**). These data suggest that patients in the IRS-high group, with low expression levels of the protective genes in the signature, are not particularly sensitive in terms of an immune response.

### Chemotherapy Responses of High- and Low-IRS Patients With Breast Cancer

**Figure 9:**
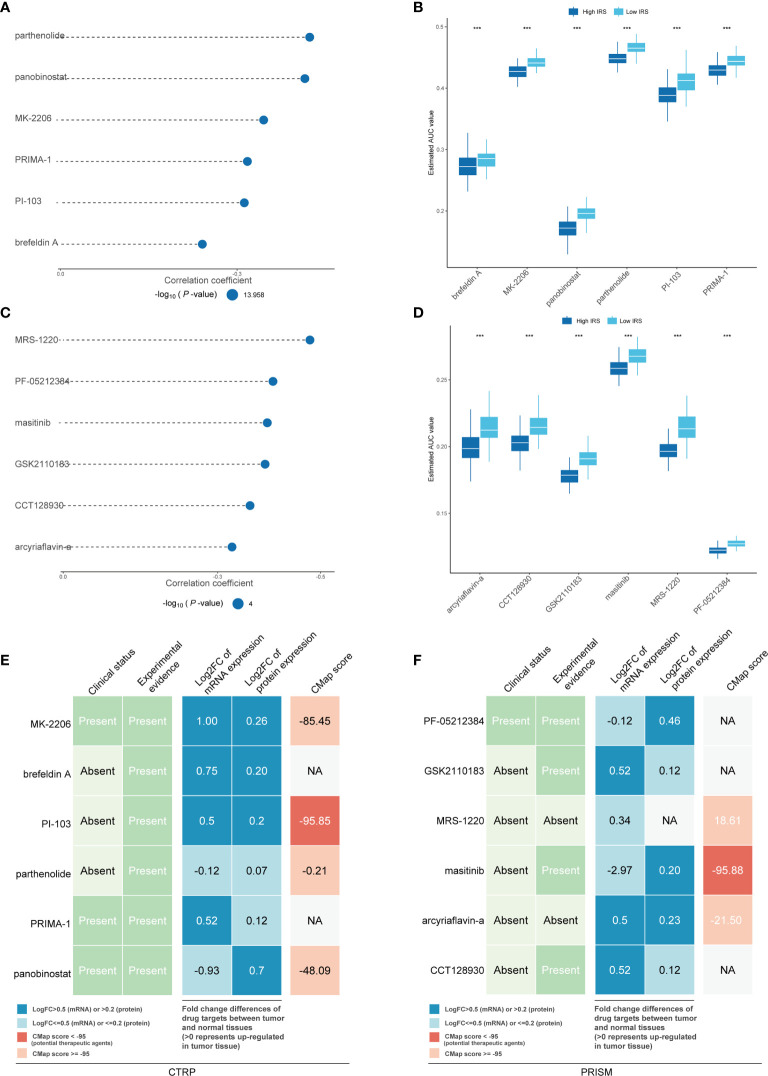
Identification of candidate agents with higher drug sensitivity in high-IRS patients. The bubble plot shows the degree of negative correlation between AUC values and IRS scores of six CTRP-derived compounds **(A)** and six PRISM-derived compounds **(C)**, with longer lines representing stronger negative correlations and implying greater drug sensitivity. The results of differential drug response analysis of CTRP-derived compounds **(B)** and six PRISM-derived compounds between the IRS-high and IRS-low groups **(D)**. Note that lower values on the y-axis of boxplots imply greater drug sensitivity. Evidence from multiple sources to identify the most promising therapeutic agents for the high-IRS group. Six CTRP-derived agents **(E)** and six PRISM-derived agents **(F)** are shown on the left and right of the diagram, respectively. *** means the p-value is less than 0.01; NA means Not Applicable.

Chemotherapy still plays an important role in breast cancer. Therefore, two different methods were used to identify drug candidates with high drug sensitivity in patients with high IRS, and two different drug response databases (CTRP and PRISM) were included. First, the AUC values and IRS were analyzed by Spearman correlation analysis to select compounds with negative correlation coefficients (Spearman r < -0.25 for CTRP or -0.30 for PRISM). Next, an analysis of the differences in drug response between the high-IRS (highest decile) and low-IRS (lowest decile) groups was performed to identify compounds with lower estimated AUC values in the high-IRS group [log2-fold change (FC) >0.05]. It is important to emphasize that a lower AUC means better drug sensitivity. Six CTRP-derived compounds (including MK-2206, brefeldin A, PI-103, parthenolide, PRIMA-1 and panobinostat) and six PRISM-derived compounds (including PF-05212384, GSK2110183, MRS-1220, masitinib, arcyriaflavin A and CCT128930) were found to be potentially sensitive in the high-IRS group. All these compounds had a negative correlation with the IRS and lower estimated AUC values in the high-IRS group (**Figures 9A, B** for CTRP and **Figures 9C, D** for PRISM).

To further explore whether the drugs we discovered have potential clinical value, multiple-perspective analyses were performed to investigate the therapeutic potential of these compounds in breast cancer. First, a thorough literature review was conducted in PubMed to identify experimental and clinical evidence of the candidate compounds in the treatment of breast cancer. Second, the log2 FC of the difference in the mRNA and protein expression levels of the genes related to the drug targets between tumor and normal tissues was calculated, and a higher log2 FC value indicated better potential for breast cancer treatment. Third, CMap analysis was used to identify compounds whose gene expression patterns were oppositional to the breast cancer-specific expression patterns (gene expression was increased in tumor tissue but decreased upon treatment with certain compounds). Two compounds, PI-103 and masitinib, had CMap scores of <-95, indicating that these compounds might have therapeutic potential for breast cancer (**Figures 9E, F** and **Supplemental Table S7**). The above methods are referenced from published literature (23).

### Building a Predictive Nomogram for Breast Cancer Patients

**Figure 10:**
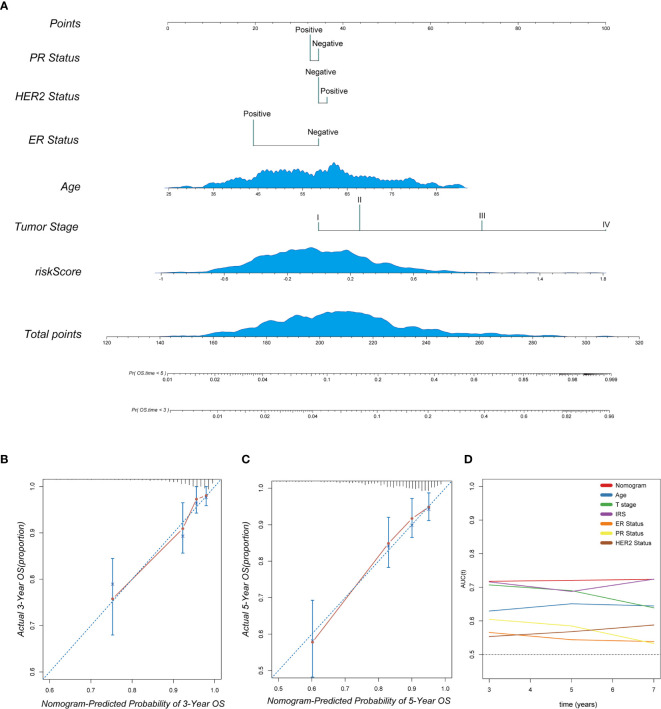
The combination of the IRS signature and clinicopathological features improves survival prediction. **(A)** A nomogram was constructed to predict 3- and 5-year OS in individual breast cancer patients. **(B, C)** Calibration analysis indicated a high accuracy of 3- and 5-year survival prediction. **(D)** tROC analysis demonstrated that the nomogram was the most stable and powerful predictor for OS among all the clinical variables.

To provide a clinically appropriate approach for predicting the OS probability of breast cancer patients, independent risk factors were used to build a risk estimation nomogram (**Figure 10A**). These predictors included age, tumor stage, PR status, HER2 status, ER status and the risk score related to immune infiltration. The calibration plots for 3- and 5-year survival probabilities in the TCGA cohort are presented in **Figures 10B, C** and suggest that the nomogram had a high level of accuracy. Furthermore, compared with other features, tROC analysis showed that the predictive nomogram was the most accurate predictor for OS, with an average AUC above 0.7 (**Figure 10D**). This means that our nomogram has good predictive value.

## Discussion

Immunotherapy is revolutionizing the treatment of multiple solid tumors (3, 4, 26, 27), and early data suggest that programmed cell death-1/programmed death ligand-1 (PD-1/PD-L1) antagonists are clinically active in a small number of patients with metastatic breast cancer (5, 28). Clinical activity seems more likely if the tumor is triple negative, PD-L1t, and/or harbors higher levels of tumor-infiltrating leukocytes (29). In metastatic triple-negative breast cancer (TNBC), atezolizumab (30) and pembrolizumab (31, 32) appear to have longer-lasting effects, suggesting that these drugs are likely to change the prognosis and lives of patients who respond to treatment. These observations indicate why the analysis of immune cells has prognostic value and why immunotherapy has become an attractive therapeutic approach. To date, several immune-related gene signatures have been developed for prognostic purposes in different types of cancers, such as ovarian cancer (33), renal cancer (34), lung cancer (35) and glioblastoma (36). Some immune-related signatures have also been reported for breast cancer, which is one of the tumors with the most accessible data (37, 38).

However, previous studies have had many unavoidable deficiencies. First, some of these immune-related gene signatures were roughly established based on literature-reported individual immune-related genes, ignoring the fact that the immune system is a cancer hallmark involving gene networks instead of several individual genes. Second, many gene signatures do not have sufficient validation groups to confirm their predictive power. Third, few established immune-related gene signatures have been integrated with the traditional prognostic system to optimize routine clinical practice. Their direct guidance for clinical work is not very effective.

To remedy these shortcomings, we have made the following changes and explorations. First, we screened the genes not from the literature reports but from the WGCNA modules based on transcriptome profiling data. This can fully ensure the interaction between the genes. Second, we used three external validation groups to ensure that our models all had good predictive power. Third, our model can predict not only the prognosis of breast cancer patients but also the response to immunotherapy. Moreover, it can also provide some suggestions for the choice of chemotherapy drugs according to the heterogeneity of tumors.

In our study, the infiltration scores of 24 immune cells were obtained from ImmuCellAI. Univariate Cox regression analysis and WGCNA identified gene modules associated with protective immune cells. The LASSO algorithm was used for promising candidates and gene establishment. Subsequently, the prognostic value of this gene signature was evaluated in the TCGA training set and in three independent external validation sets. The risk score derived from the immune-related gene signature is called the IRS in our study. In the meta-analysis and subgroup analysis, the IRS still had the capacity to discriminate high-risk patients. Then, we analyzed the clinicopathological features and predicted the response to immunotherapy of patients in different risk groups. We also predicted the choice of chemotherapeutic drugs based on the tumor heterogeneity of patients in different risk groups. Finally, a nomogram was built based on the IRS and other clinicopathological variables to quantify the risk assessment and survival probability of breast cancer patients. In brief, our data suggest that low expression levels of protective genes and high expression levels of risk genes give patients a higher IRS, and patients in the high-risk group are not very sensitive to immunotherapy.

Some biomarkers involved in our gene signature have been reported in many cancers, but most of them have rarely been investigated in immune infiltration. For example, BCL2 L14 (BCL2-like 14) is a well-reported gene associated with apoptosis. Among its related pathways are TP53, which regulates the transcription of cell death genes (39). GBP2 is also associated with apoptosis, and the upregulation of GBP2 is associated with neuronal apoptosis in the rat brain cortex following traumatic brain injury (40). Surprisingly, the most statistically significant protective gene, FAM159A, is sparsely reported in academia and has great research potential. FAM92B, which looks similar to FAM159A, has been mainly reported in inflammatory bowel disease. Rioux *et al.* reported a genome-wide association study confirming FAM92B as a Crohn’s disease susceptibility gene (41). For SYTL3, the only risk biomarker in our study, its knockdown enhanced the amount of bacillus Calmette-Guérin (BCG) within tumors and its suppressive effect on tumor growth (42). Among the 15 genes in the signature, there were also some biomarkers related to immunity. The chemokine family genes CCL1 and CCR9 have been reported to be associated with the recruitment of regulatory T cells (Tregs) (43, 44). MS4A1, the gene encoding the B cell surface marker CD20, is significantly downregulated in human colorectal carcinoma (45). TAPBPL has been reported to interact preferentially with MHC I in the absence of glycosylation (46). However, none of these reports have provided solid evidence for a direct relationship between these genes and immune infiltration. In summary, the biological functions associated with the tumor immune infiltration of the novel gene signature still need further investigation in breast cancer.

It is also worth mentioning that the expression of a single gene may be different in different data sets. This may be caused by different sequencing platforms or different samples. Therefore, every gene chip has its own specificity. It is very undesirable to use the expression of a single gene to make predictions. However, gene signature can remedy this problem. Multigene verification can reduce the bias caused by the specificity of a single gene.

In our prediction of potential drugs, we were surprised to find two potential compounds, PI-103 and masitinib. Both compounds have CMap scores less than -95, which means that these compounds might have therapeutic potential for breast cancer. PI-103 is a potent PI3K and mTOR inhibitor that exhibits antiproliferative properties in a panel of human cancer cell lines (47). Recent studies have shown that the combination of anthracyclines and PI-103 can impair the PI3K pathway, increasing DNA damage and thus reducing the growth of breast cancer cells (48). Masitinib is a potent, orally bioavailable, and selective inhibitor of c-Kit. It also inhibits PDGFRα/β, Lyn, Lck, and, to a lesser extent, FGFR3 and FAK. Masitinib has antiproliferative and proapoptotic activity and low toxicity (49–51). Because of its inhibition of PDGFR, mastinib has been found to inhibit the luminal type of breast cancer (52). In addition to its antitumor effects, masitinib is also useful in Alzheimer’s disease. This part of the study is already in phase III clinical trials (NCT01872598). Unfortunately, both compounds lack sufficient clinical data to demonstrate that they can be used in the clinical treatment of breast cancer.

Undeniably, there are still some limitations in our study. First, this study lacks validation of laboratory data and clinical data, so the assessment of the prognostic value and the possibility of the clinical application of immune-related gene signatures need to be further validated in larger prospective trials. Second, further experimental studies are needed to elucidate the specific immune-related biological functions of these 15 genes. Finally, because of the significant immunological (and clinical prognostic) differences between patients with metastatic and nonmetastatic breast cancer, careful consideration needs to be given to the population to which our signature applies. To minimize this bias, we included ER, PR and HER2 status in the final nomogram to seek the best predictive effect.

In summary, we established a novel immune-related gene signature to predict the prognosis of breast cancer patients and provide clinicians with a new reference for treatment. According to our predictive model, patients with low expression levels of protective genes and high expression levels of risk genes will obtain a higher IRS, and patients in the high-risk group are not very sensitive to immunotherapy. Additionally, we predicted some chemotherapeutic agents that may be more sensitive in the high-risk group. Integrating this information with clinicopathological features, we constructed a nomogram to quantify the risk assessment of individual patients. The immune-related gene signature-based model could be a useful tool to select high-risk patients who may benefit from immunotherapy and thus facilitate the personalized management of breast cancer.

## Data Availability Statement

The original contributions presented in the study are included in the article/**Supplementary Material**. Further inquiries can be directed to the corresponding author.

## Author Contributions

YP and HY conceived the study, conducted most of the bioinformatics data analysis and drafted the manuscript together. YJ and FQ were involved in screening for immune-related genes and constructed the PPI network. HR and ZT reorganized the original data from the public database and searched the literature. YZ, CQ and BZ participated in part of the data analysis and figure production. SL guided the entire analysis process, determined the direction of the research for each section, and made detailed revisions to the manuscript. SL was responsible for raising the research funds. All authors contributed to the article and approved the submitted version.

## Conflict of Interest

The authors declare that the research was conducted in the absence of any commercial or financial relationships that could be construed as a potential conflict of interest.

## Supplementary Material

Supplementary Figure 1 The figure of the soft threshold power by WGCNA.

Supplementary Figure 2 GO analysis of genes in the red module.

Supplementary Figure 3 Correlation analysis between protective immune cells and IRS in TCGA and METABRIC cohorts.

Supplementary Figure 4 Results of the clinicopathological feature univariate and multivariate Cox regression analyses of OS in the luminal subtype of breast cancer in TCGA database.

Supplementary Figure 5 Results of the clinicopathological feature univariate and multivariate Cox regression analyses of OS in the basal-like subtype of breast cancer in TCGA database.

Supplementary Figure 6 Results of the clinicopathological feature univariate and multivariate Cox regression analyses of OS in the HER2 subtype of breast cancer in TCGA database.
