# Supplementary figures and images for: Construction and Validation of an Immune Infiltration-Related Gene Signature for the Prediction of Prognosis and Therapeutic Response in Breast Cancer
Source: Front Immunol. 2021 Apr 27;12:666137. doi: 10.3389/fimmu.2021.666137 (PMC8110914; doi:10.3389/fimmu.2021.666137)

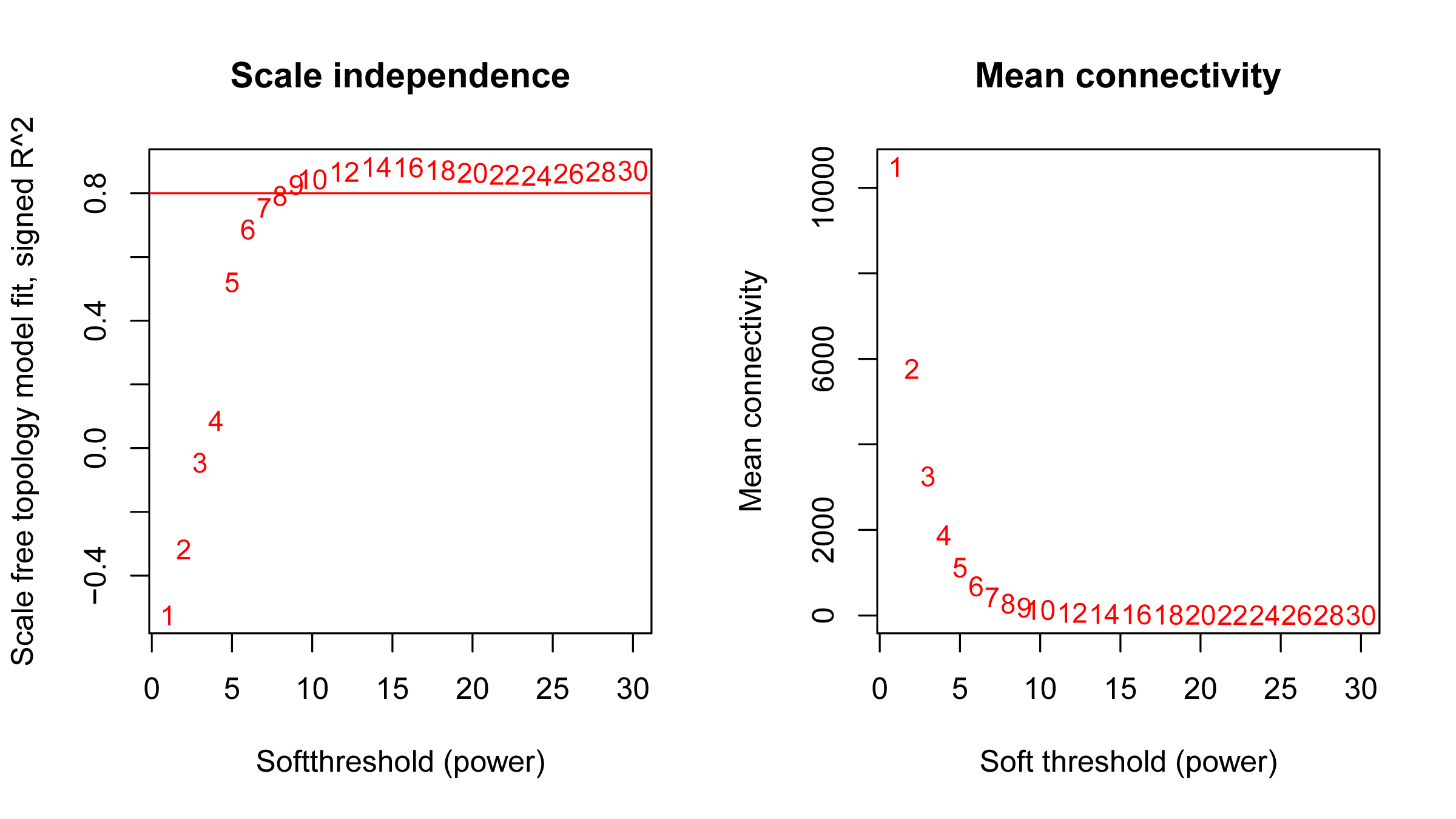

Supplement: Supplementary Figure 1 — The figure of the soft threshold power by WGCNA. [file Image_1.tif]

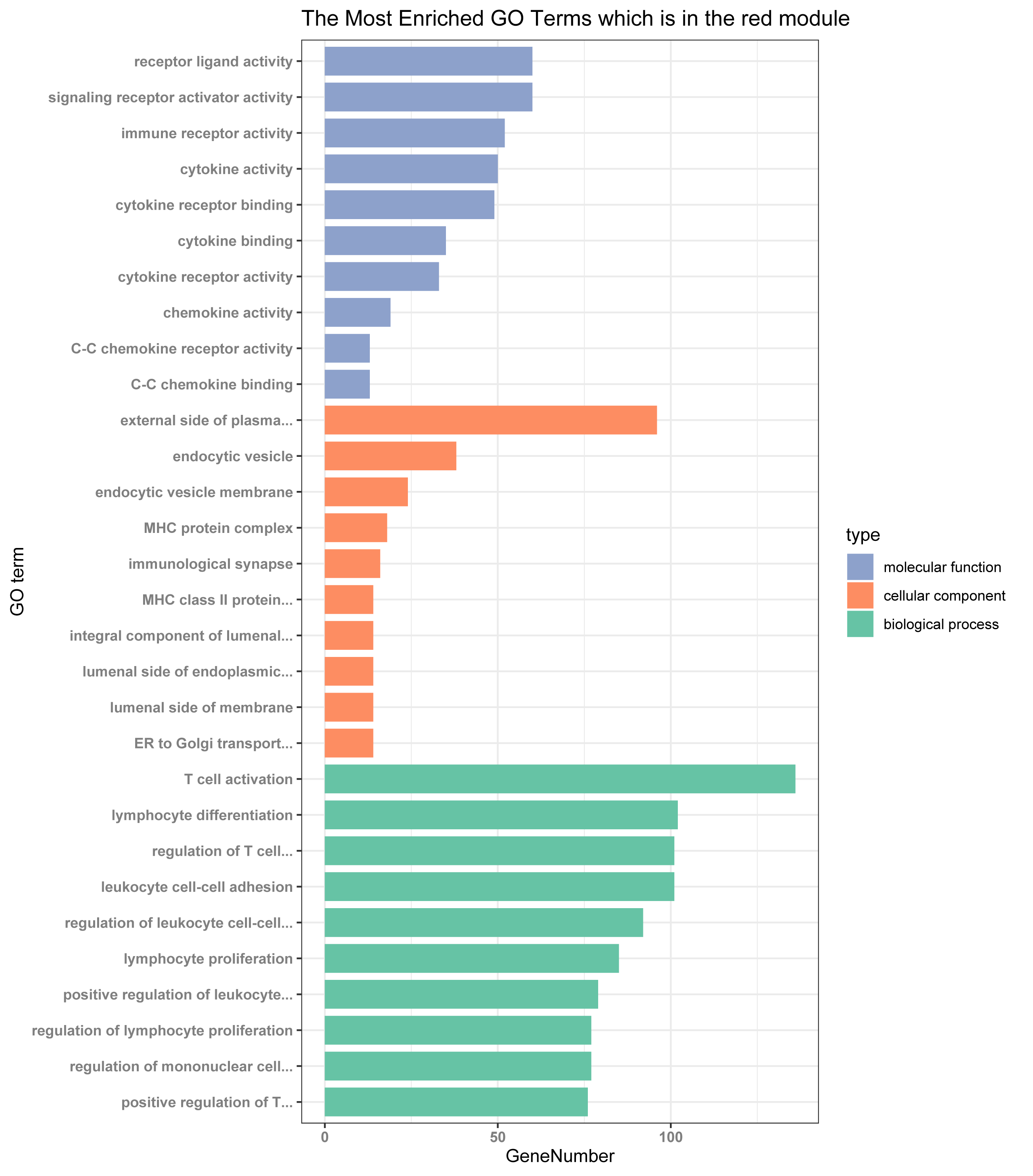

Supplement: Supplementary Figure 2 — GO analysis of genes in the red module. [file Image_2.tif]

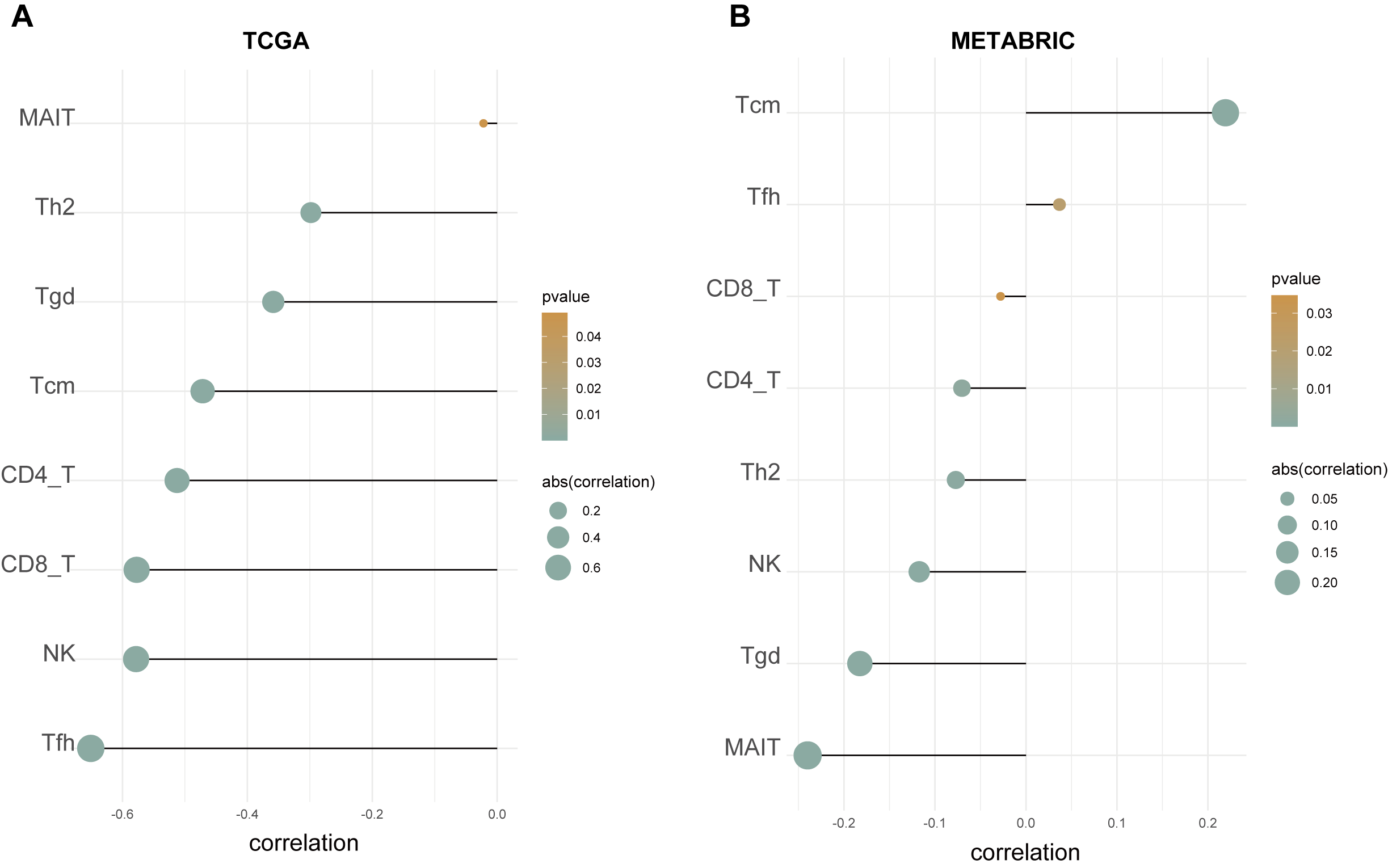

Supplement: Supplementary Figure 3 — Correlation analysis between protective immune cells and IRS in TCGA and METABRIC cohorts. [file Image_3.tif]

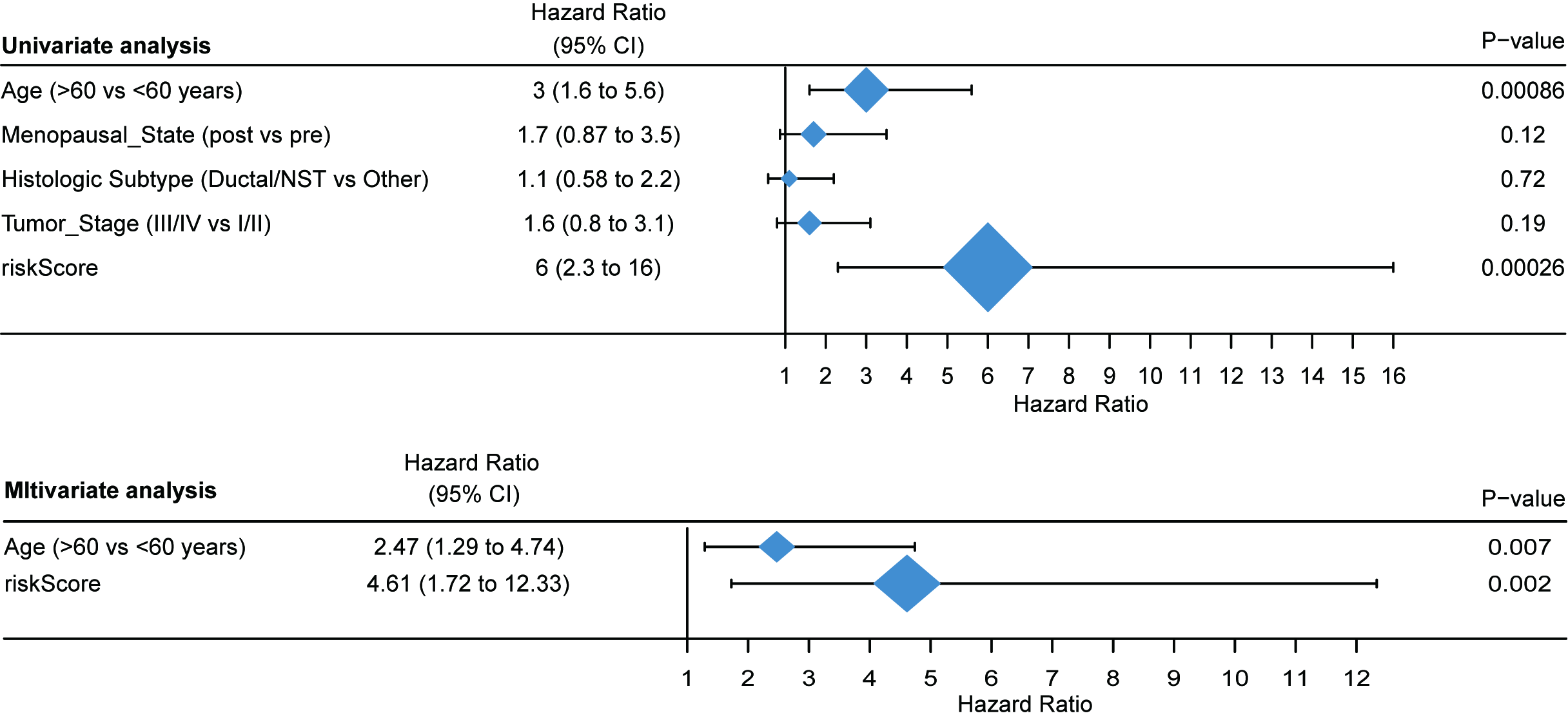

Supplement: Supplementary Figure 4 — Results of the clinicopathological feature univariate and multivariate Cox regression analyses of OS in the luminal subtype of breast cancer in TCGA database. [file Image_4.tif]

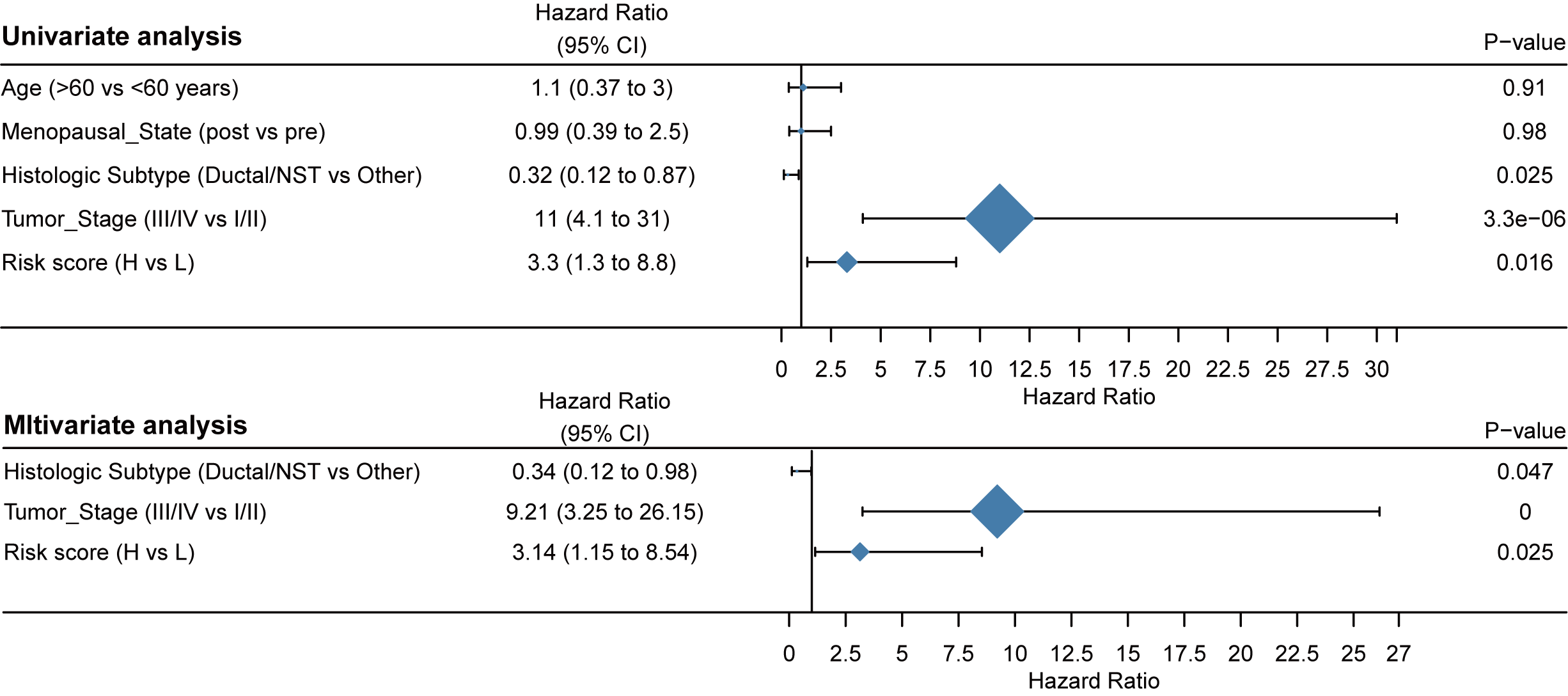

Supplement: Supplementary Figure 5 — Results of the clinicopathological feature univariate and multivariate Cox regression analyses of OS in the basal-like subtype of breast cancer in TCGA database. [file Image_5.tif]

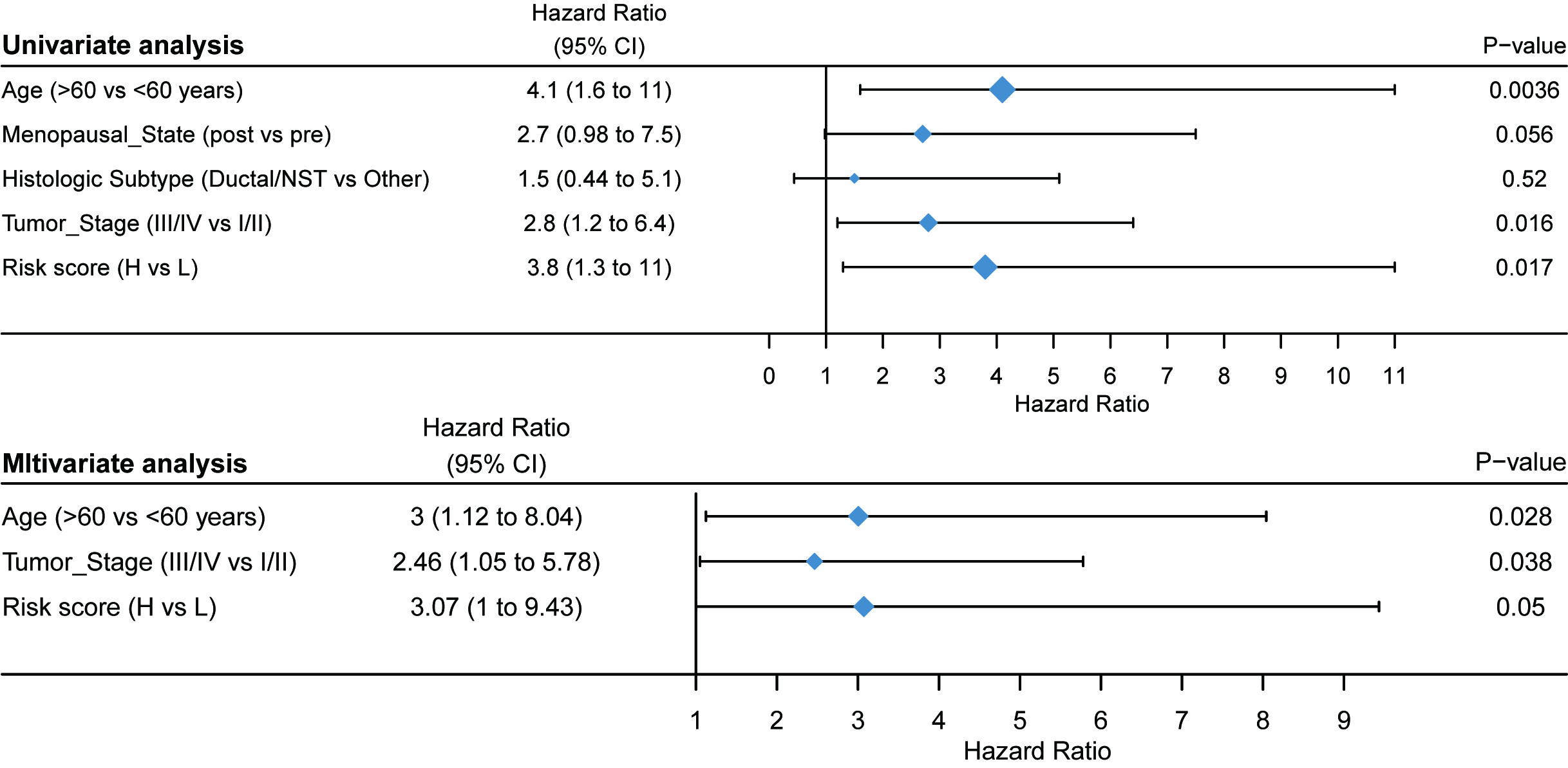

Supplement: Supplementary Figure 6 — Results of the clinicopathological feature univariate and multivariate Cox regression analyses of OS in the HER2 subtype of breast cancer in TCGA database. [file Image_6.tif]
