# Supplementary material for: Construction and Validation of an Immune Infiltration-Related Gene Signature for the Prediction of Prognosis and Therapeutic Response in Breast Cancer
Source: Front Immunol. 2021 Apr 27;12:666137. doi: 10.3389/fimmu.2021.666137 (PMC8110914; doi:10.3389/fimmu.2021.666137)
Supplement: Supplementary file 7 [file DataSheet_1.pdf]

Table S1. The clinical information distribution between METABRIC and TCGA cohort

	METABRIC (N=1978)	TCGA (N=1007)	P-value
**Age**			<0.001
Mean (SD)	61.1 (13.0)	58.4 (13.2)	
Median [Min, Max]	61.8 [21.9, 96.3]	59.0 [26.0, 90.0]	
**Menopausal_State**			<0.001
Post	1553 (78.5%)	642 (63.7%)	
Pre	424 (21.4%)	352 (35.0%)	
NA	1 (0.1%)	13(1.3%)	
**His_Subtype**			0.00255
Ductal/NST	1489 (75.3%)	714 (71.4%)	
Other	445 (22.5%)	280 (28.3%)	
Missing	44 (2.2%)	13 (1.3%)	
**Histologic_Grade**			<0.001
1	168 (8.5%)	0 (0%)	
2	771 (39.0%)	0 (0%)	
3	952 (48.1%)	0 (0%)	
NA	87 (4.4%)	1007 (100%)	
**Tumor_Stage**			<0.001
I	500 (25.3%)	173 (17.3%)	
II	825 (41.7%)	570 (57.0%)	
III	118 (6.0%)	222 (22.2%)	
IV	10 (0.5%)	17 (1.7%)	
Missing	525 (26.5%)	25 (2.5%)	
**OS.time**			
Mean (SD)	125 (76.0)	42.6 (38.5)	<0.001
Median [Min, Max]	117 [1.23, 355]	30.2 [1.01, 283]	
**OS**			
Alive	835 (42.2%)	863 (85.7%)	<0.001
Dead	1143 (57.8%)	144 (14.3%)	
